# Does individual learning styles influence the choice to use a web-based ECG learning programme in a blended learning setting?

**DOI:** 10.1186/1472-6920-12-5

**Published:** 2012-01-16

**Authors:** Mikael Nilsson, Jan Östergren, Uno Fors, Anette Rickenlund, Lennart Jorfeldt, Kenneth Caidahl, Gunilla Bolinder

**Affiliations:** 1 Department of Medicine, Karolinska University Hospital Solna, Karolinska Institutet, 171 76, Stockholm, Sweden; 2 Department of Computer and Systems Science (DSV), Stockholm University, Forum 100 S-164 40 Kista, Sweden; 3 Department of Molecular Medicine and Surgery, Karolinska University Hospital Solna, Karolinska Institutet, 171 76, Stockholm, Sweden

## Abstract

**Background:**

The compressed curriculum in modern knowledge-intensive medicine demands useful tools to achieve approved learning aims in a limited space of time. Web-based learning can be used in different ways to enhance learning. Little is however known regarding its optimal utilisation. Our aim was to investigate if the individual learning styles of medical students influence the choice to use a web-based ECG learning programme in a blended learning setting.

**Methods:**

The programme, with three types of modules (learning content, self-assessment questions and interactive ECG interpretation training), was offered on a voluntary basis during a face to face ECG learning course for undergraduate medical students. The Index of Learning Styles (ILS) and a general questionnaire including questions about computer and Internet usage, preferred future speciality and prior experience of E-learning were used to explore different factors related to the choice of using the programme or not.

**Results:**

93 (76%) out of 123 students answered the ILS instrument and 91 the general questionnaire. 55 students (59%) were defined as users of the web-based ECG-interpretation programme. Cronbach's alpha was analysed with coefficients above 0.7 in all of the four dimensions of ILS. There were no significant differences with regard to learning styles, as assessed by ILS, between the user and non-user groups; Active/Reflective; Visual/Verbal; Sensing/Intuitive; and Sequential/Global (p = 0.56-0.96). Neither did gender, prior experience of E-learning or preference for future speciality differ between groups.

**Conclusion:**

Among medical students, neither learning styles according to ILS, nor a number of other characteristics seem to influence the choice to use a web-based ECG programme. This finding was consistent also when the usage of the different modules in the programme were considered. Thus, the findings suggest that web-based learning may attract a broad variety of medical students.

## Background

The time available to teach the medical curriculum is shortening due to expanding knowledge, and incorporation of new educational modules aiming to a broader competence [1]. The electrocardiogram (ECG) remains an indispensable tool in the practice of clinical medicine [2].

Understanding ECG and training its interpretation is a continuing challenge for both students and medical professionals [2,3]. However, the optimal way to learn and train ECG interpretation needs to be studied [4]. Pedagogical strategies for teaching ECG and what learning media to use vary from dance [5] on one extreme, to computer assisted learning (CAL)[6].

We have constructed and started to evaluate a web-based ECG learning programme designed for undergraduate medical students. In an initial study, the medical students ranked the Web-based ECG-interpretation programme as a useful instrument to learn ECG, and performance in a diagnostic test improved with use of the programme [7]. Our experience tells us that approximately 60% of the students use the programme when offered to them without cost, and thus about 40% do not.

We do not know why some students prefer to learn ECG on the web, while others do not.

However, another study on first year medical students showed that 69-84% of the participants preferred to learn from a book rather than from a web-based Learning Management System [8]. Link and Martz have pointed out that E-Learning must be appropriate to the students' level of computer expertise in order not to become a source of frustration [9].

It is still not known why students choose to use online learning materials or not.

The aim of this study was to investigate whether individual learning styles or other characteristics affect the students' choice to use the web-based ECG learning programme.

## Methods

The study was approved by the local ethics committee at the Karolinska Institutet (KI).123 medical students attending the traditional ECG-training course (lectures and seminars) at two educational sites at the Karolinska University hospital were offered to use the web-based ECG learning programme as an additional learning method. Due to a change in the medical education curriculum at KI the students of this population had reached two different semesters, the fourth and fifth, at the two sites studied (Solna and Huddinge respectively). However, all students had the same course objectives. In the beginning of the course the students received a short introduction to the web-based ECG learning programme, and were free to use the programme in whatever way and to the extent that they wished at any time during the course. The traditional ECG training includes compulsory lectures and seminars of about ten hours. All students have previously participated in another course on the electrophysiology behind the ECG, but had not been given any training in the interpretation of ECGs.

### The ECG learning program

The development and structure of the web-based ECG-interpretation programme have been described earlier [7]. In summary, the programme is designed to be able to serve as a complement to the standard ECG education, or to be used as a "stand-alone" tool for self-regulated learning. The system contains three types of modules: Learning content (text, pictures and animation); self-assessment questions, and an interactive ECG interpretation training section. The programme is easily accessed via the learning management system used by KI (Ping Pong™). The log-in credentials are the same as to access the e-mail system at KI, thus facilitating the access to the system. User activity in terms of accessed modules and time spent in the different modules was automatically logged by Ping Pong.

### Learning styles

Learning styles can be defined as characteristic preferences for alternative ways of taking in and processing information [10]. There are several instruments constructed to assess learning style [11]. In this study we used the Index of Learning Styles (ILS) by Felder and Silverman [12,13]. The reasons for this choice were several, including that the instrument is scientifically accepted and relatively well studied [14-17]. The ILS instrument is also seen as time efficient with 44 questions and it is free of charge in a non commercialised setting. Further on, studies have found ILS to be acceptable in terms of reliability and validity [18-20].

The ILS provides a separate score for each of four dimensions (active-reflective, visual-verbal, sensing-intuitive, and sequential-global). The scores in each dimension range from +11 to -11 in steps of 2. For a detailed description of ILS and the four dimensions we refer to the original article by Felder & Silverman [13].

The instrument was translated into Swedish after approval from Dr Felder. The translation was controlled by two reviewers (JÖ and UF) who translated the questions backwards from Swedish to English and was compared with the original instrument which led to small adjustments in the Swedish version to attain congruence.

At the end of the ECG-training course the students were asked to fill in the ILS instrument and a general questionnaire. The latter included questions about computer and Internet usage in general, as well as a ranking of the pedagogic value of the three different learning modules of the ECG programme (learning content, self assessment, interpretation training), preference for future medical speciality and estimation of prior experience of E-learning.

In their original classification Felder and Silverman used three categories to define a learning style in each of the four dimensions. 1-3 +/-, indicates a fairly well balanced style on that scale. 5-7 +/-, indicates a moderate preference for one dimension of the scale suggesting that the student will learn more easily in a teaching environment which favours that dimension. 9-11+/-, implies a very strong preference for one dimension of the scale. The student at the extreme of the scale may have a real difficulty learning in an environment which does not support that preference [12].

We used a modification of this approach which has been previously used [21].

**Figure 1:**
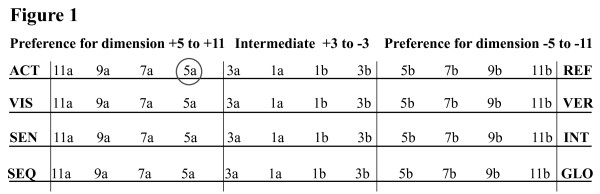
**shows how the preference of each dimension is determined, depending on the score that locate it on the respective scales**. For example a student who answer "yes" to eight and "no" to three questions out of the eleven representing the active-reflective dimension would score 5a (marked with grey ring) and regarded having a preference for active learning style in this dimension.

In this modification the four dimensions of learning styles were each divided into three categories instead of five. The intermediate category had a score between +3 to -3 and values above and below this was defined as a preference for the respective learning style in that dimension, as depicted in Figure 1.

### User activity

To facilitate the analysis of the user activities of the ECG-system, an active "user" was defined as a student who had been logged on for at least 30 minutes to the system.

### Statistical analysis

To analyse categorical data the Chi square test was used. If the number of students was less then five in a group Fisher's exact test was used. Numerical data where analysed using Mann-Whitney U-test. We used SPSS 17.0 for the statistical analysis above. Cronbach's alpha was calculated for each dimension of the ILS using SAS^® ^System 9.1. The level of statistical significance was set to p < 0.05.

## Results

### General findings

**Figure 2:**
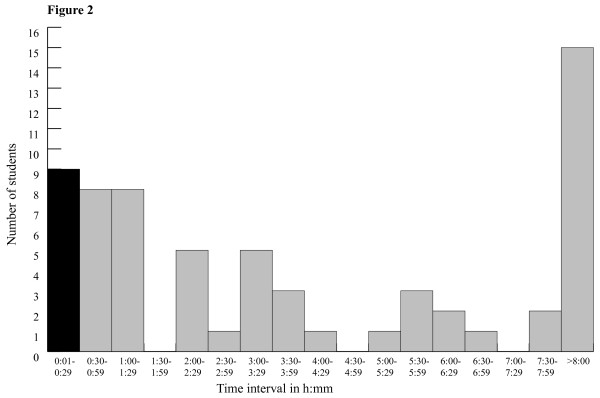
**shows the number of students who logged into the programme and the time using it**. Students who logged in but not met the definition of a user (30 min or more) are marked in black.

All students in the study had computer access at home. In the user group 98%, and in the nonuser group 92% visited internet sites daily. Ninety-three (76%) out of the 123 students answered the Index of Learning Styles instrument and 91 (74%) the general questionnaire. 55 (59%) out of 93 students were defined as users of the web-based ECG-system. User activity, as measured by total time, is displayed in Figure 2.

There was no significant difference in demographic characteristics or learning styles between the students at the two participating sites. Therefore, all students were gathered as one single group in the analyses performed. Background characteristics of the total study population are summarized in Table 1 where it can be seen that none of the demographic variables influenced the level of use.

**Table 1:** 

	User n	User %	Non User n	Non User %	*p*
**Female gender**	29	53%	21	55%	*p = 0.81*

**Male**	26	47%	16	45%	

**Internet connection home**:					*p = 0.19*
No	1	2%	1	3%	
Modem	1	2%	4	11%	
Broadband	53	96%	32	86%	

**Internet usage**:					*p = 0.18*
Daily	53	98%	34	92%	
Couple of times during a week	1	2%	3	8%	
Sometimes a week	0		0		
Seldom	0		0		
Never	0		0		

**Computer at home:**					
Yes	54	100%	37	100%	
No	0				

**I master computer usage**					*p = 0.29
(for my educational needs)					
1 (do not agree)	0		0		
2	1	2%	1	3%	
3	3	6%	0		
4	2	4%	4	11	
5	19	37%	18	49%	
6 (completely agree )	27	52%	14	38%	

Table 1 shows the students' background characteristics. The number of responses varies because not all respondents answered all questions. *p *refers to Chi^2 ^except for *Mann-Whitney U-test.

### Learning styles and level of usage

There were no significant differences between the user and non-user groups according to learning styles: Active/Reflective p = 0.53. Visual/Verbal p = 0.66. Sensing/Intuitive p = 0.96 Sequential/Global p = 0.86. Nor were there any differences when the result was analysed in relation to usage of the three types of modules (learning content, self-assessment questions and interactive ECG interpretation). These results are summarized in Table 2.

**Table 2:** 

	User n	User %	Non User n	Non User %	*Chi2 p*
**Total**	55	59%	38	41%	

**Learning styles**					

**Active/Reflective**					*p = 0.53*

Active	9	16%	9	24%	

Intermediate Act/Ref	38	69%	22	58%	

Reflective	8	15%	7	18%	

**Visual/Verbal**					*p = 0.66*

Visual	24	44%	15	39%	

Intermediate Vis/Ver	29	53%	20	53%	

Verbal	2	4%	3	8%	

**Sensing/Intuitive**					*p = 0.96*

Sensing	23	42%	15	39%	

Intermediate Sen/Int	27	49%	19	50%	

Intuitive	5	9%	4	11%	

**Sequential/Global**					

Sequential	10	18%	8	21%	*p = 0.86*

Intermediate Seq/Glo	31	56%	22	58%	

Global	14	25%	8	21%	

**Prior experience to E-learning**					*p = 0.30*

Yes	44	81%	26	72%	

No	10	19%	10	28%	

**Future most interesting speciality**					*p = 0.17*

Internal/Family med	12	34%	4	15%	

Operating	16	46%	12	46%	

Indecisive	7	20%	10	39%	

Table 2 show the result compared the user and non user group divided into learning style. The user was defined as a student who logged on for at least 30 minutes to the system. P value for Chi^2 ^comparison between user and non-user according to the respective dimensions. The number of responses varies because not all respondents answered all questions.

Moreover, there were no significant differences between the user and non-user groups according to prior experience of E-learning and future preferred choice of speciality.

A further analysis of the data revealed that learning styles did not influence the level of usage of the three different components of the ECG, system (Learning content, Self -assessment questions and Interactive ECG interpretation). The results of this analysis is shown in Table 3.

**Table 3:** 

	Learning content		Self-assessment questions		Interactive ECG interpretation	
	Mean ± SD		Mean ± SD		Mean ± SD	

**Students ranking the pedagogic value of the modules in the web-based programme 1-6 (worst possible -best possible)**	4.5 ± 1.2		4.5 ± 1.3		5.0 ± 1.3	

	Median (IQR)		Median (IQR)		Median (IQR)	

**Time spent in modules (hh:mm)**	01:21 (0:06-05:30)		0:13 (0:03-00:42)		0:37 (0:04-02:55)	

**Total n**	User n*	**Non User n**	User n**	**Non User n**	User n***	**Non User n**

	40	53	58	35	33	60

	**User %**	**Non User %**	**User %**	**Non User %**	**User %**	**Non User %**

**Total %**	43%	57%	62%	38%	35%	65%

**Learning styles**						

**Active/Reflective**						

Active	18%	21%	15%	26%	12%	23%

Intermediate Act/Ref	70%	60%	71%	54%	67%	63%

Reflective	13%	19%	14%	21%	21%	13%

**Visual/Verbal**						

Visual	50%	36%	44%	38%	39%	43%

Intermediate Vis/Ver	48%	57%	51%	56%	55%	52%

Verbal	3%	8%	3%	9%	6%	5%

**Sensing/Intuitive**						

Sensing	38%	43%	42%	38%	48%	37%

Intermediate Sen/Int	55%	45%	48%	51%	45%	52%

Intuitive	8%	11%	8%	12%	6%	12%

**Sequential/Global**						

Sequential	18%	21%	17%	24%	24%	17%

Intermediate Seq/Glo	58%	57%	55%	60%	55%	58%

Global	25%	23%	27%	18%	21%	25%

The upper part of the table 3 shows the students ranking, time spent, with inter quartile range (IQR). The lower part results in user - non user from the individual modules divided into learning style. *A user in the Learning content module was defined as a student who logged on for at least 20 min in that specific module. **A user in the Self-assessment questions module was defined as a student who logged on for at least 10 min in that specific module. ***A user in the Interactive ECG interpretation module was defined as a student who logged on for at least 20 min in that specific module.

Cronbach's alpha was analysed according to learning styles with the following scores: Active/Reflective 0.77, Visual/Verbal 0.84, Sensing/Intuitive 0.86, Sequential/Global 0.81.

### Ranking of the educational benefits

The users were asked to rank the educational benefits of the learning modules based on a six-graded scale (1- poor to 6 - very useful). On average the students ranked "Learning content" 4.5 (SD 1.2), "Self-assessment questions" 4.6 (SD 1.1), and "Interactive ECG interpretation training" 5.0 (SD 1.3).

### User definition

In order to test if our definition of users could have affected the results we analysed the influence of different cut off levels used to define a user (30, 60 or120 minutes). The results did not differ depending on user definition; Active/Reflective p = 0.53-0.76-0.93, Visual/Verbal p = 0.66-1.0-0.74, Sensing/Intuitive p = 0.96-0.79-0.82, Sequential/Global p = 0.86-0.30-0.32 (p for 30-60-120 minutes respectively).

## Discussion

Two reviews have investigated learning styles with special focus on health science education. Romanelli stresses the lack of a conceptual framework for both learning style theory and measurement and conclude that faculty members should make concentrated efforts to teach in a multi style fashion [14]. Cook concludes that further research in web-based learning could clarify the feasibility and effectiveness of assessing and adapting to learning [15]. Others have criticized the use of learning styles as predictors of learning preferences with the argument that there are more important factors involved in the learning process. Some work has been done since Cooks review was published, but there are only a few papers relevant for medical web-based education [16,21-23]. Further, the findings of these studies were not consistent and it is thus still not known why students prefer to use online learning materials or not.

In this study we found no evidence supporting that students' learning styles, according to ILS, influence the choice to use the web-based ECG-interpretation programme or not in a blended learning setting. This result is in accordance with those of Cook et al who found no association between ILS scores and different web-based format preferences in medical residents [11]. However, other studies indicate other possibilities. For example, McNulty, studying medical students, collected entry logs for two different web-based applications (a discussion forum and a tutorial). It was found that students with "Sensing" preference tended to use the web-based applications to a larger extent than the ones with an "Intuitive" preference. Further, differences in the usage of web-based applications for the "Perceiving/Judging" dimension in the instrument were also found. Another learning style instrument, the Mayers-Briggs type indicator (MBTI) was used in that study, but an association between the sensing dimension in MBTI and ILS has been described suggesting that these two learning styles indices bear a close resemblance to each other for this dimension.

Even when using one specific instrument, different types of format in the evaluating questionnaires (i e forced-choice preference or Likert scale) may influence the ability to compare the results between studies. Using a self report survey Brown et al came to the conclusion that the learning styles of health science students, as measured by the ILS, can be used, although to a limited extent, as a predictor of students' attitudes towards E-learning. Both Brown and Cook primarily used indirect measures assessed on an Online Learning Environment Survey (OLES) and an end of course questionnaire using a scale ranging from 1-6 as preference. Thus, they did not use recorded activity in the programme as a denominator, as was done in the present study.

An indirect measure, asking students forced-choice preference items, was used by Johnson & Johnson. They found a statistical difference for college students in the active -reflective dimension. Active students preferred face-to-face study groups rather than online study groups, but online quizzes rather than pencil and paper quizzes. However, Johnson & Johnson used the individual result in the four ILS dimensions as a continuous variable and compared the group average instead of the more commonly used scored categorisation [24]. Their approach lend support by the finding of Cook, that up to one third of learners change style classification although the mean score change is not large [19].

Blended learning can be defined in a broader way as a combination of face-to face instruction and computer-mediated instruction [25].

However the blend can be of different categories and using a blended learning approach could mean that the web-based component of the course needs to have a specific instructional design to match the personal learning style of the individual student [25].

In this study we have defined the blended learning setting as the voluntary opportunity for a student to use web-based learning as a complement to the traditional teaching.

However, in the setting of a self-directed stand-alone web-based course learning styles might have affected the usage differently. A study using ILS found a difference between a self-directed version and a collaborative version of an online course [26]. In that study significant associations between students' learning styles and success in distance education were found, suggesting a relationship between learning style and ability to respond to web-based learning.

The inconsistency in research results regarding learning styles and their relation to different studying preferences could also relate to differences between studied groups of students (e g different academic level). The Swedish admission system for medical studies is mainly based on high grades and on expected high theoretical academic performance. It might be assumed that these students have a high capacity to adapt their studies to different learning situations. This may affect the ability to generalize our results to other groups of students.

The concept of learning styles in pedagogical research has been criticised from different perspectives. Massa and Mayer state that there are instruments that correctly distinguish between verbalizers and visualizers. However, data do not provide support for the idea that different instructional methods should be used for these groups [27]. Others have reviewed the field and conclude that an impact of learning style theory on teaching and learning efficiency is unproven by current empirical work [11]. However the same authors also recognise that the learning styles theories may still be of importance to pedagogy; personalized education and students' self-awareness (learning to learn).

The results of Cronbach's alpha analysis with a coefficients above 0.7 in all of four dimensions is comparable to others, and indicate that the Swedish translation of the ILS is usable in this study.

One potential bias of our study could be that the definition of a user could be incorrect. To test for this we performed a sensitivity analysis using three different user time cut-offs with the same result. Thus it is not likely that the definition of users have influenced our results.

Another potential limitation is the sample size. The pre-study power analysis used data from a pilot study measuring mean usage time of the web-base course according to learning styles which indicated a need for 8 persons in each group. Based upon this we calculated that a sample size of 60-100 persons was needed to achieve a power of 80% to detect a difference in usage time according to learning style at the p < 0.05 level. A post-hoc analysis with the achieved dichotomous data indicated that 12 persons were needed in each group to achieve 80% power to detect a 50% relative difference in learning style between users and non-users.

In order to preserve statistical power the four dimensions of learning styles were each divided into three categories of learning style (according to Cooke) instead of five (as described by Felder). This modification increased the number of students in each group with a preference for a certain learning style and should thus if anything enhance the possibility to find differences with the actual sample size.

Out of the 123 students, 76% answered the ILS instrument and 73% the general questionnaire. A fairly good proportion of the students thus participated in the study. In the studied group 59% were defined as users. Using data from Ping Pong the number of users of the web-based ECG learning programme among the group not answering the ILS-instrument was only 10. Thus, we believe that possible differences between participants and non-participants in the survey did not affect the results of our study to a substantial degree.

Other factors than learning style may influence the choice to use the web-based ECG learning programme or not. Our experience from discussions with students is that time seems to be an important factor. To spend time to learn to use a fairly new medium, not knowing the effectiveness, may be an obstacle in applying web-based learning. Time for studies without computers exist in different environments, for example on the bus, in bed or in non-computerised areas within the university. The social situation of individual students could affect the time to spend on the Internet as a medium.

## Conclusion

Among medical students, neither learning styles according to ILS, nor a number of other characteristics seem to influence the choice to use a web-based ECG programme.

In consistency with the results from Cook and Mayer this study supports the conclusion that medical educators need not presently take learning styles into account for instructional adaptations of web based learning. Our results support web-based learning as a suitable alternative learning tool for most medical students, regardless of learning style or other characteristics.

## Competing interests

MN is a shareholder in the company which has developed the web-based ECG-interpretation programme. There are no other conflicts of interest in relation to this manuscript.

## Authors' contributions

All authors participated in designing the study on the initiative of MN, who managed all practical parts of the study and drafted the manuscript. GB UF and JÖ participated in finalising the manuscript. AR, LJ and KC had minor comments and approved the final manuscript.

## Pre-publication history

The pre-publication history for this paper can be accessed here:

http://www.biomedcentral.com/1472-6920/12/5/prepub
